# Tracking Flow in Real Time: Continuous Measurement of Game‐Induced Flow in Virtual Reality

**DOI:** 10.1111/psyp.70283

**Published:** 2026-04-07

**Authors:** Sura Genc, Elif Surer, Marc Wittmann, Tzvetan Popov, Bigna Lenggenhager

**Affiliations:** ^1^ Department of Psychology University of Konstanz Konstanz Germany; ^2^ Graduate School of Informatics, Middle East Technical University Ankara Türkiye; ^3^ Institute for Frontier Areas of Psychology and Mental Health Freiburg Germany; ^4^ Department of Psychology University of Zurich Zurich Switzerland; ^5^ AIR–Association for Independent Research Zurich Switzerland

**Keywords:** flow experience, heart rate variability, real‐time measurement, time perception

## Abstract

Flow experience is characterized by becoming deeply absorbed in a task, by losing track of time, and diminished self‐awareness. Psychological flow research typically involves the qualitative or quantitative assessment of flow states after the experience with questionnaires. This approach is limited, as post‐task reports provide an overall assessment of the whole experience, but lack the temporal resolution required to analyze together with psychophysiological data and fail to capture flow as it unfolds in real time. We introduce a novel method that enables the continuous and real‐time subjective measurement of flow. Forty participants indicated their perceived degree of flow by pressing a custom‐made foot pedal (i.e., real‐time report) while they engaged in a flow‐inducing activity (i.e., playing the video game *Thumper* in virtual reality). They played the game under two conditions, one with the pedal and one without, while electrocardiogram (ECG) signals were recorded. After each condition, participants completed the validated Flow Short Scale (FSS) as a post‐task report. Results support the hypotheses that interacting with the pedal did not interfere with: (a) participants' flow experience as measured with the FSS, (b) heart‐rate variability (HRV), and (c) the pedal provided a reliable self‐assessment of flow, as indicated by the correlation between real‐time and post‐task flow ratings. HRV analyses revealed evidence for associations between the high‐frequency (HF)‐HRV, the low‐frequency (LF)/HF ratio, and perceived flow, suggesting that parasympathetic activity and autonomic balance may play a role in the flow experience. In conclusion, this proposed novel empirical method enables assessment of the temporal evolution of a flow experience and its potential links to psychophysiological indicators extending beyond HRV.

## Impact Statement

We present a novel, non‐disruptive method for simultaneously collecting continuous subjective assessments of the flow experience, and accompanying psychophysiological responses. This approach addresses two key limitations in flow research: the lack of continuous subjective data and thus its temporal misalignment with physiological measures. Our findings show that the method enables valid, reliable tracking of the temporal dynamics of the flow, offering new insights into the relationship between subjective experience and physiological, and potentially neural, states.

## 1 Introduction

The flow experience is defined as an optimal mental state characterized by deep absorption in a task accompanied by a sense of complete involvement in and enjoyment of the process (Csikszentmihalyi 1990). According to Csikszentmihalyi (1990), achieving flow requires an optimal skill‐challenge balance, as well as clearly defined goals and immediate feedback. Individuals in a flow state perceive their actions as effortless and fluent, often lose track of time (Khoshnoud et al. 2022), and experience reduced bodily and spatial awareness (Peifer and Tan 2021) and diminished self‐awareness (Sadlo 2016). A flow state not only enhances performance on tasks (Engeser and Rheinberg 2008) but also heightens well‐being (Nakamura and Csikszentmihalyi 2002; Weber et al. 2016; Kühn et al. 2018).

Perceived flow is typically assessed through self‐reports (i.e., questionnaires) after the experience. Early research used the Experience Sampling Method (ESM), where participants received random prompts throughout the day and completed forms about their current activity, mood, cognitive efficiency, and motivation (Csikszentmihalyi and Larson 2014). This method showed that flow most often occurs when individuals are fully engaged in activities that provide a balance between a high degree of challenge and appropriate skills. The Flow State Scale (Jackson and Marsh 1996) and the Flow Short Scale (Rheinberg et al. 2003) both validated tools to measure flow across a range of settings, were developed on this basis. These self‐reports have since been widely used to characterize the emotional and cognitive components of the flow experience (Kivikangas 2006; Castellar et al. 2016; de Manzano et al. 2010; Ulrich et al. 2016).

Flow research aims to explore bodily and neurocognitive dynamics by identifying the physiological markers that accompany its subjective characteristics (Khoshnoud et al. 2020). A common approach to identify physiological correlates of flow involves recording physiological data (Katahira et al. 2018; Huskey et al. 2018; Tozman et al. 2015; Khoshnoud et al. 2022; de Manzano et al. 2010) during a flow‐inducing task followed by an assessment of perceived flow using self‐reports, referred to here as post‐task reports. These post‐task reports serve as proxies for the participants' subjective flow experiences, and physiological states are recorded for a wider contextual understanding. However, this approach faces two methodological challenges and constraints.

The first challenge is that attempting to measure flow experience over the course of the task disrupts the immersive state, making it difficult to capture it in real time. This paradoxical aspect has led researchers to measure flow using retrospective methods, such as post‐task reports or experience‐sampling techniques, which still capture experiences only after they have occurred (Jackson and Marsh 1996). These methods, although useful, depend on participants' retrospective evaluations (Peifer et al. 2014) rather than capturing the flow state as it unfolds in real time.

Secondly, post‐task reports lack the continuous nature of recorded states (Khoshnoud et al. 2020) required for a simultaneous analysis with physiological real‐time data. This temporal mismatch restricts integration of subjective experience with the physiological measurements.

**FIGURE 1:**
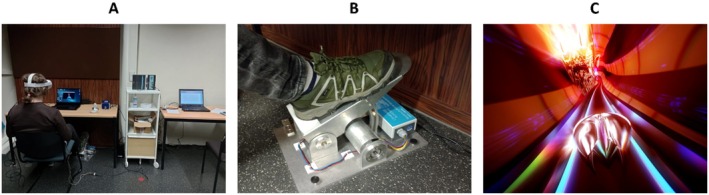
Setup of the pedal. (A) Experimental setup showing a participant wearing the HMD and ECG electrodes while playing the game. The data‐collection computer and the experimenter's workspace are positioned on the right. (B) Custom‐designed pedal. (C) Thumper game interface, Drool LLC 2013–2019.

The study described here addressed these methodological constraints by introducing a novel method to achieve a continuous, real‐time subjective assessment of flow. Participants continuously indicated their perceived degree of flow by using a custom‐made foot pedal (see Figure 1) during the task, a process referred to as a real‐time report. Within this framework, fully pressing the pedal with the foot, represented by the highest recorded pedal angle, indicated a high degree of flow experience. Conversely, reducing pressure on the pedal, resulting in a lower recorded pedal angle, signified a decrease in the individual's flow level. The use of the pedal enabled participants to report their flow levels during the flow experience simply and intuitively. This approach also enabled the simultaneous collection, analysis, and correlation of psychophysiological and continuous subjective data throughout a flow‐inducing task.

Various tasks have been used to investigate flow, including playing a video game (Rutrecht et al. 2021), engaging in sports (Sinnett et al. 2020), and performing music (de Manzano et al. 2010). Although it is difficult to find a task that will allow people to experience flow in a laboratory environment, video games have become a widely used method, as they provide clear goals and immediate feedback, and the difficulty level of the games can be adjusted according to the person's skills (Chanel et al. 2011; Khoshnoud et al. 2022; Nacke and Lindley 2008). Here we used the video game, *Thumper*, as a flow‐inducing task that has been previously successfully utilized (Rutrecht et al. 2021).

The participants played Thumper in virtual reality (VR) under two conditions (25 min each): one with the pedal (pedal condition) and one without (control condition). In the pedal condition, participants were instructed to continuously modulate pressure on the pedal to report real‐time variations in their degree of flow during gameplay. In the control condition, they played the game without using the pedal. Electrocardiogram (ECG) signals were recorded during both gameplay sessions. After each session, participants completed several questionnaires, including the Flow Short Scale (FSS), the Subjective Time, Self, Space Scale (STSS), the Self‐Assessment Manikin Scale (SAM), also completed prior to the task, and the Altered States of Consciousness Rating Scale (ASC).

To test the validity of our new method, we addressed three methodological research questions: whether using the pedal interferes with the flow experience, whether the pedal provides a reliable real‐time measure of flow, and whether using the pedal affects physiological responses.

Our first research question examined whether using the pedal to report the current flow level interferes with participants' flow experience, which is a common concern. Given the pedal's user‐friendly and intuitive design and its compatibility with gaming gestures, we anticipated that participants would experience a similar level of flow under both conditions. We hypothesized that there would be no considerable difference between the pedal and control conditions (Hypothesis 1a) in participants' post‐task flow scores measured by the FSS.

Assuming the pedal would not interfere with participants' flow experiences, we expected similar perceptions of time across the two conditions. We also hypothesized that there would be no considerable difference across conditions in perceived duration, frequency of thinking about time, and the speed of time passage (Hypothesis 1b) as measured by the STSS.

We further hypothesized that bodily awareness would remain consistent across conditions (Hypothesis 1c) as assessed by the STSS. We expected that valence and arousal levels would be similar across conditions (Hypothesis 1d) as evaluated by the SAM. Finally, reflecting aspects of self‐consciousness, we hypothesized that disembodiment, insightfulness, and blissfulness scores would be similar (Hypothesis 1e) as measured by the ASC. These hypotheses reflect the idea that the pedal would not disrupt any of the participants' flow‐related experiences.

The second research question investigated whether the pedal provided a reliable real‐time flow measure by examining its relationship with post‐task flow scores. We hypothesized that there would be a positive correlation between real‐time flow ratings collected via the pedal and post‐task flow scores measured by the FSS (Hypothesis 2). Testing these hypotheses would show whether the custom‐designed pedal can serve as a valid tool for the real‐time self‐assessment of flow.

The third research question examined whether using the pedal to provide real‐time flow assessment interferes with physiological responses, specifically the activation of the autonomic nervous system (ANS) observed through HRV. Researchers investigated the balance between the sympathetic nervous system (SNS) and the parasympathetic nervous system (PNS) activity during flow by examining heart‐rate variability (HRV) patterns (Harmat et al. 2015; Tozman et al. 2015; de Manzano et al. 2010), with the SNS linked to arousal and the PNS promoting relaxation and recovery. The high‐frequency (HF) component of HRV is widely recognized as an indicator of parasympathetic or vagal activity (Shaffer et al. 2014). While the low‐frequency (LF) component has traditionally been linked to cardiac sympathetic neural activity (Montano et al. 1994), it has recently been shown to be influenced by both parasympathetic and sympathetic activity (Thomas et al. 2019).

Given its established role in reflecting ANS activity, we used HRV metrics to assess the physiological effects of our method. We hypothesized that there would be evidence for the null hypothesis concerning average HF‐HRV (Hypothesis 3a) and average LF‐HRV (Hypothesis 3b) across conditions, indicating that the pedal does not interfere with ANS activity.

As an exploratory analysis, we first investigated the relationship between real‐time flow ratings and HRV metrics across five 5‐min epochs to explore the interaction between perceived flow experiences and ANS activity. A cross‐correlation between real‐time flow ratings and the inter‐beat interval (IBI) data over five 5‐min epochs was also performed to investigate the reported increase in sympathetic activity during flow (de Manzano et al. 2010; Bian et al. 2016).

Testing these hypotheses would determine whether the pedal‐based method is a suitable approach for investigating the subjective experience of flow in conjunction with physiological responses.

## 2 Method

### 2.1 Participants

Forty‐four participants participated in the study. Since the experiment included a virtual reality (VR) game, we had to halt the experimental sessions for four participants who reported feeling unwell, leading to a final sample of 40 (29 women, *M*_age_: 22.78, SD_age_: 5.22). All participants preferred to use the pedal with their right foot. They had normal or corrected‐to‐normal vision.

Gaming motivations were assessed using the items of the Gamer Motivation Profile test (Quantic Foundry, n.d.). Thirty‐six of the participants were casual gamers, meaning they played video games infrequently or only in short sessions, while four were core/mid‐core gamers, meaning they played regularly, but were not highly competitive or deeply invested in the gaming culture. None of the participants were hardcore gamers, defined as highly dedicated players who engage in gaming seriously or competitively. Participants had an average gaming level of 1.45 ± 0.90, indicating that they spent 0 to 1 days playing video games for at least 30 min in a typical week. Thirty‐nine participants had no prior experience with the game Thumper.

Participants were recruited through the University of Konstanz's SONA platform, where current studies at the university are advertised. Experimental sessions took place in the Cognitive Psychology Laboratory at the University of Konstanz. Written informed consent was obtained prior to participation in the experiment. All experimental procedures were approved by the Ethics Committee of the Faculty of Sciences of the University of Konstanz (approval number: 37/2022).

### 2.2 Materials

Participants were equipped with a head‐mounted display (HMD), ECG electrodes, a game controller, and the foot pedal. The HMD used was the Oculus Quest 2, which has a resolution of 1832 × 1920 pixels per eye and a 90‐Hz refresh rate. A SteelSeries Stratus Duo wireless controller was used for gameplay. While the HMD and ECG electrodes were worn throughout all tasks, the pedal was used only during the pedal condition. Participants were allowed to take short breaks between tasks, during which they could remove the HMD.

#### 2.2.1 Pedal

The pedal was custom‐designed and built by the Scientific Workshop of the University of Konstanz specifically for the purposes of this study. It records real‐time angle measurements ranging from 0° to 32.4°; it provides a resolution of 0.1° with a maximum recorded value of 324 corresponding to full depression of the pedal (Figure 1). Continuous pedal output was recorded with a 90‐Hz sampling rate.

The pedal was used to collect the real‐time report of flow from participants. Fully pressing the pedal indicated a high state of flow, while complete decompression signaled the absence of flow. This process allowed participants to continuously report their flow levels, enabling the collection of time‐series data on perceived flow across the entire task.

#### 2.2.2 Video Game: Thumper

Thumper has proven successful in inducing flow (Khoshnoud et al. 2022; Rutrecht et al. 2021). Developed and released by Drool (2016), it is a rhythm‐action game played through a third‐person perspective from above a moving beetle, where the player braces against obstacles with simple button‐press controls.

Players guide the beetle through a vibrant, roller‐coaster type environment at a consistent pace throughout the game. They anticipate approaching obstacles and turns using the designated buttons on the game controller while maneuvering through the track. The game immerses players in their experience by dynamically integrating synchronized visual and auditory elements after each interaction with obstacles, thereby enhancing the overall gameplay immersion. Players can synchronize their actions with the rhythm of the sounds to enhance their reaction time to obstacles, turns, and rewards. The game's objective is to maximize one's score in each level and section by striking as many rewards as possible while avoiding collisions with obstacles and turns (Figure 1). We used the Steam platform to access and launch the game.

#### 2.2.3 Physiological Recording Equipment

The ECG measures the electrical activity of the heart through small sensors attached to the skin. We used Biopac MP150 System hardware and AcqKnowledge 4.1 software for data acquisition. Continuous ECG signals were recorded with a 1000‐Hz sampling rate and band‐pass filtered within the 0.01–120‐Hz range. The ECG signal was acquired using three electrodes positioned according to the lead II Einthoven configuration: two active electrodes placed on the right clavicle and the left rib, and a ground electrode placed on the left clavicle. DocCheck adhesive electrodes (diameter = 50 mm) were attached.

#### 2.2.4 Questionnaires

All questionnaires described below were presented via Qualtrics (Qualtrics, Provo, UT), an online survey and experience management platform hosted in the cloud.

The FSS (Flow Short Scale) was used to measure perceived flow during the two gaming sessions. We did not use The Flow State Scale (Jackson and Marsh 1996), as it was developed to assess flow in sports and physical activities rather than gaming contexts. The FSS developed by Rheinberg et al. (2003; English version: Rheinberg et al. 2023) consists of 16 items, including fluency, absorption, worry (i.e., perceived importance), and skill‐challenge balance components. The first ten items were used to compute the total flow score in this study.

Absorption refers to the deep engagement or total concentration that individuals experience when they are in flow. Individuals often lose track of time during absorption and become unaware of environmental distractions. It is measured by four items in the FSS (e.g., “I feel just the right amount of challenge”. and “I am totally absorbed in what I am doing”). Fluency, known as the smoothness of performance, refers to the seamless and effortless execution of tasks while in flow. When individuals experience fluency, they perceive their actions as fluid and natural, with a sense of control over their performance. It is measured by six items in the FSS (e.g., “My thoughts/activities run fluidly and smoothly.” and “I have no difficulty concentrating”).

The STSS (The Subjective Time, Self, and Space Questionnaire) consisting of five items is used for assessment of bodily awareness and time perception (Jokic et al. 2018). The initial two items assessed the intensity of participants' bodily and spatial experiences during gameplay. Responses were recorded on a 7‐point Likert scale accompanied by non‐verbal pictorial representations.

The remaining three items evaluated participants' perceptions of time during the two gaming sessions. Participants were asked to rate the duration of the gaming session, the frequency of their thoughts about time, and the speed of time passage. Responses were indicated by adjusting a slider ranging from ‘not at all’ to ‘extremely intense’.

The SAM (Self‐Assessment Manikin) questionnaire assesses the changes in valence and arousal levels on a 5‐point Likert scale with non‐verbal pictorial representations of the human body (Bradley and Lang 1994). We used this scale both before and after each session to measure changes in valence and arousal from the beginning to the end of each session.

The 5D‐ASC (Five‐Dimensional Altered States of Consciousness Rating Scale; referred to here as ASC) is an instrument designed to assess various dimensions of unusual subjective experiences (Dittrich et al. 2006; Dittrich et al. 2010). It consists of 94 items grouped into 11 subscales, three of which were used in this study: disembodiment, insightfulness, and blissful state. Participants rate their experiences on a slider ranging from “No, not more than usual” to “Yes, much more than usually,” indicating the extent to which they perceived each experience during a specific task. Participants completed this scale after each session.

### 2.3 Procedure

**FIGURE 2:**
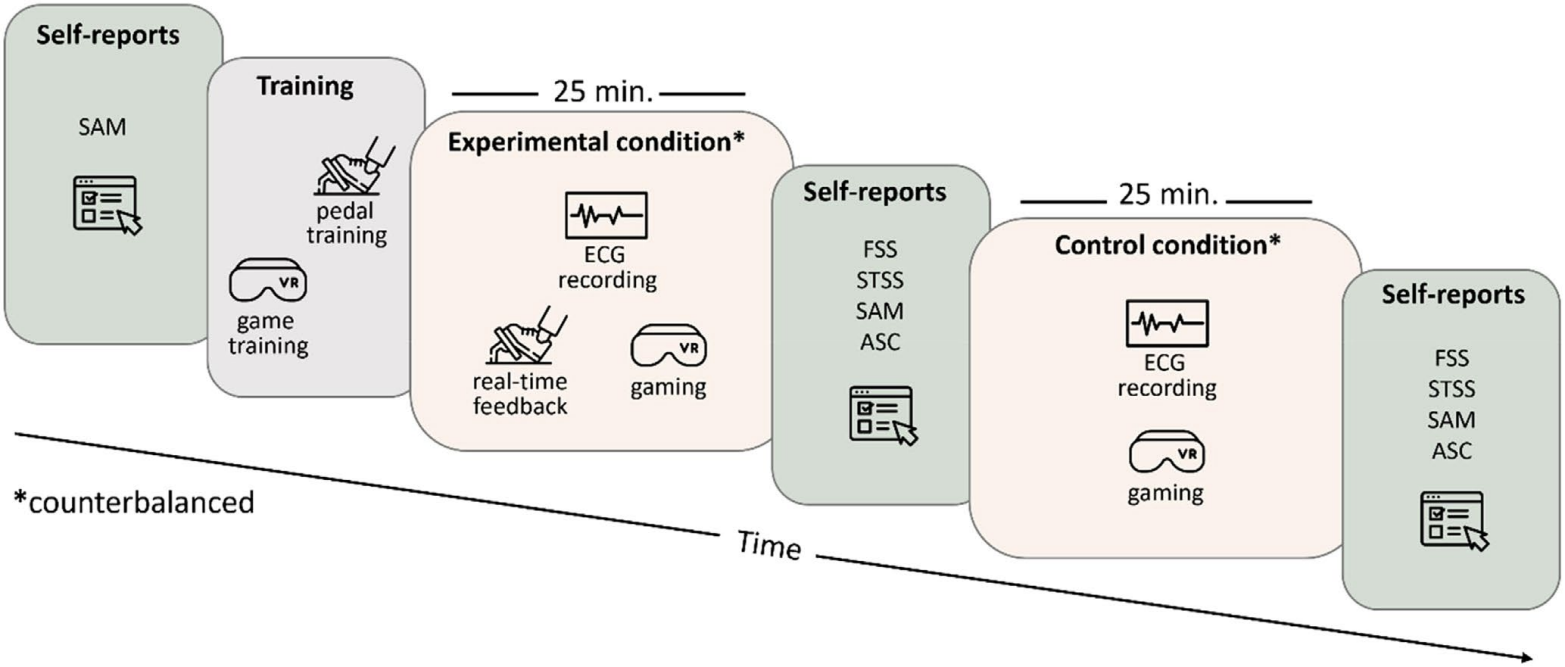
Overview of the study protocol. The experimental procedure, lasting approximately 100 min, consisted of four stages: introduction, training, and two gaming sessions (counterbalanced). After briefing and completing surveys, participants underwent training with the game Thumper and the pedal. They then played the game in virtual reality under two conditions, one with the pedal and one without, while their ECG signals were recorded. After each condition, they completed the Flow Short Scale (FSS), the Subjective Time, Self, Space Scale (STSS), the Self‐Assessment Manikin Scale (SAM; also completed prior to the task), and the Altered States of Consciousness Rating Scale (ASC). Icons made by Iconjam, Freepik, azmianshori and Nur syifa fauziah from www.flaticon.com.

The experimental procedure lasted approximately 100 min per participant and included four stages: introduction, training, and two gaming sessions presented in counterbalanced order (Figure 2). Participants were briefed on the study details and the concept of flow upon arrival at the laboratory. Any questions were addressed to ensure a clear understanding of the flow experience. Participants then signed the informed consent form and completed demographic surveys along with the SAM questionnaire.

Participants then proceeded to a training phase, which consisted of separate sessions for the game and the pedal, each lasting approximately 5 min. During the game training, participants played Thumper in practice mode until they reached sub‐level 1.10 (i.e., the tenth stage of level one). In the pedal‐training session, they practiced pressing and releasing the pedal in sync with the vertical feedback slider displayed on the screen, allowing them to become familiar with the pedal's range of motion. They were also encouraged to adjust the pedal's position to ensure comfort before the main task.

They were then fitted with ECG electrodes and the HMD. Each participant completed two 25‐min game sessions in a counterbalanced order: one with the pedal and one without. In the experimental condition, participants placed their preferred foot (39 participants preferred their right foot) on the pedal while gaming, pressed it when they sensed flow, and adjusted the pressure accordingly. They were encouraged to report their level of flow continuously using the pedal as often as possible. In the control condition, they played the game without using the pedal. After each session, they completed the same set of post‐task questionnaires: the FSS, STSS, SAM, and ASC. ECG signals were recorded throughout both sessions, and pedal output was recorded during the pedal condition.

Participants then removed the HMD and electrodes and provided feedback on task difficulty and experiences by answering open‐ended questions. They received a compensation of 10 EUR per hour.

### 2.4 Data Processing and Analysis

#### 2.4.1 Processing Behavioral Data

The preprocessing of questionnaires and pedal data was conducted in Python. Continuous pedal data were collected and processed at a sampling rate of 90 Hz. Raw data were extracted from the time interval between the first and last trigger signals for each participant, ensuring alignment with ECG signals recorded using the same triggers. The cleaned pedal data were then segmented into five 5‐min epochs per participant, and the mean pedal angle was computed for each epoch.

Flow scores were calculated from the first ten items in the FSS (7‐point Likert scale) for all participants (*N* = 40). Absorption scores were calculated using items 1, 2, 6, and 10, and fluency scores using items 2, 4, 5, 7, 8, and 9. Perceived duration (in seconds) was calculated with items 3 and 4 from the STSS. Ratings for thinking about time and the perceived speed of time passage were determined with items 5 and 6 from the STSS. Bodily awareness was derived by averaging the first two items in the STSS.

Emotional valence and arousal changes were computed as the difference between baseline and post‐task scores from the SAM (items 1 and 2, respectively). Three subscales from the 5D‐ASC were calculated as the mean of corresponding items: disembodiment (items 1, 2, and 3), insightfulness (items 4 and 5), and blissful state (items 7, 8, and 9). The data from the FSS and the STSS were then visualized using the ptitprince package in Python (Allen et al. 2021).

#### 2.4.2 Processing ECG Signals

ECG data (*N* = 37) were preprocessed, and HRV metrics were computed using the NeuroKit2 package in Python (Makowski et al. 2021), which provides the functions for biosignal processing. The preprocessing pipeline included down‐sampling, signal cleaning, IBI calculation, and the extraction of HRV indices. The ECG signals were down‐sampled from 1000 Hz to 250 Hz using the Fourier method (implemented via signal‐resample in NeuroKit2). They were then preprocessed using Hamilton (2002)'s method (implemented via ecg‐clean in NeuroKit2).

Frequency‐domain metrics of HRV were calculated with Welch (2003)'s method of spectral‐density estimation implemented via hrv‐frequency in NeuroKit2 within 5‐min epochs. All datasets were standardized to *z*‐scores implemented via the StandardScaler function from the sklearn.preprocessing module.

#### 2.4.3 Statistical Analyses

The sample size (*N* = 44) was determined based on the literature (Khoshnoud et al. 2022). Four participants with incomplete recordings were excluded from the HRV data analysis, resulting in a final sample size of 40.

A predefined Bayes factor of 3 was set as the criterion for interpreting evidence strength, with a Bayes factor (BF_10_) of < 0.33 (i.e., BF_01_ > 3) considered as moderate evidence for the null hypothesis and a Bayes factor (BF_10_) of > 3 considered as moderate evidence for the alternative hypothesis.

To test Hypothesis 1, Bayesian *t*‐tests were performed to compare post‐task flow scores (1a), STSS scores (1b, 1c), SAM scores (1d), and ASC scores (1e) between the two conditions using JASP (JASP Team 2025). Priors were defined using JASP's default Cauchy prior (*r* = 0.707), which was adopted given the absence of prior empirical estimates of effect sizes for this novel pedal‐based paradigm. Sensitivity analyses were conducted using a narrower Cauchy prior (*r* = 0.1) to assess the robustness of conclusions to prior width.

To test Hypothesis 2, a Bayesian correlation analysis was performed between the post‐task flow scores from the pedal condition and the five epochs of real‐time flow ratings. This analysis was implemented via the Pingouin package in Python (Vallat 2018).

To test Hypothesis 3, Bayesian *t*‐tests were conducted comparing HRV metrics (3a and 3b) across conditions. These analyses were also implemented in JASP using the default Cauchy prior (*r* = 0.707), with additional sensitivity analyses conducted using *r* = 0.1.

#### 2.4.4 Exploratory Analyses

As part of our exploratory analysis, we conducted a cross‐correlation between HRV metrics (LF and HF power and LF/HF ratio) and real‐time flow ratings. HRV metrics were computed using the NeuroKit2 module across 25 sliding windows, each with a 5‐min duration and a 1‐min step size, enabling HRV estimation at 60‐s intervals. To ensure HRV metrics were also available within the first 5 min, we adapted the windowing scheme to include progressively increasing segments (0–60, 0–120, 0–180, 0–240, and 0–300 s) followed by standard 5‐min sliding windows (e.g., 60–360, 120–420, etc.). HRV metrics and pedal data for each window were standardized to z‐scores. We excluded the initial 60‐s window from the analysis, as it falls below the recommended 120‐s lower limit (Shaffer and Ginsberg 2017) for valid frequency‐domain HRV estimation.

We used the statsmodels package in Python (Seabold and Perktold 2010) to implement the cross‐correlation. Each lag unit in the cross‐correlation represented a 60‐s shift. We adopted the method used by Basgol et al. (2025) to quantify the evidence levels. At each lag, we conducted a Bayesian *t*‐test (using the pingouin module) to compare the observed group‐level mean correlation to zero, which provided Bayes Factors (BF_10_). This analysis was implemented via the Pingouin package in Python (Vallat 2018).

A linear mixed‐effects model was also conducted using 24 windows of dataset to examine whether LF‐HRV, HF‐HRV, and their interaction predict pedal real‐time flow ratings (using the statsmodels package in Python; Seabold and Perktold 2010).

Another cross‐correlation between real‐time flow ratings and IBI over five 5‐min epochs was performed to further examine the relationship between subjective flow experiences and ANS activity. IBIs were calculated from the heart rate (HR) (IBI = 60,000/HR). To preprocess IBIs, we used the procedure from Denk et al. (2024): IBIs were cleaned by removing values with a relative change greater than 35% from one sample (using the interpolate function CubicSpline module) and then interpolated using a cubic spline. To enable time‐alignment, both pedal and IBI data were down‐sampled to 4 Hz using different methods based on their respective characteristics. The pedal data was downsampled by rounding timestamps to the nearest 0.25 s and averaging all values within each bin. The IBIs were resampled at 0.25‐s intervals during the cubic spline interpolation. Each lag unit in the cross‐correlation represented a 0.25‐s shift. We implemented the cross‐correlation and assessed the evidence with the procedure described above.

## 3 Results

### 3.1 Post‐Task Flow Scores

**FIGURE 3:**
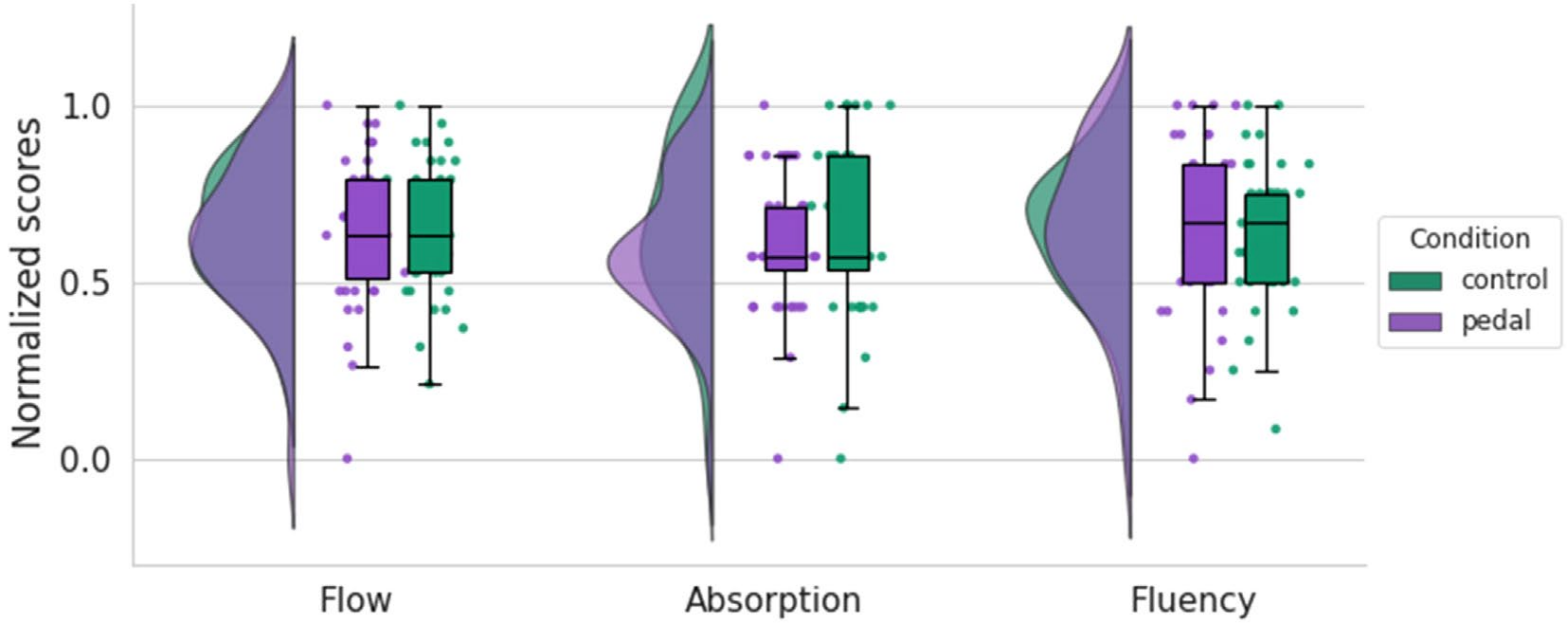
Descriptives of post‐task flow scores in control and pedal conditions. Normalized scores from the Flow Short Scale (FSS) subscales are displayed across two experimental conditions: control (green) and pedal (purple). Each panel includes a density plot, individual data points, and a boxplot representing the distribution of normalized scores.

Participants reported an average flow score of 4.91 (SD = 1.16) in the pedal condition and a flow score of 5.00 (SD = 1.04) in the control condition (Figure 3). We observed moderate evidence that the pedal did not affect participants' perceived flow across conditions (BF_01_ = 5.15, 95% CI [−0.22, 0.37]), supporting Hypothesis 1a. Bayes factors also moderately favored the null hypothesis for absorption scores (BF_01_ = 3.18, 95% CI [−0.13, 0.47]) and for fluency scores (BF_01_ = 5.85, 95% CI [−0.31, 0.29]). Under the narrower prior specification (Cauchy *r* = 0.1), *t*‐test continued to favor the null hypothesis for post‐task flow scores (BF_01_ = 1.47), absorption (BF_01_ = 1.19), and fluency (BF_01_ = 1.55), albeit with weaker evidence in the anecdotal range.

**FIGURE 4:**
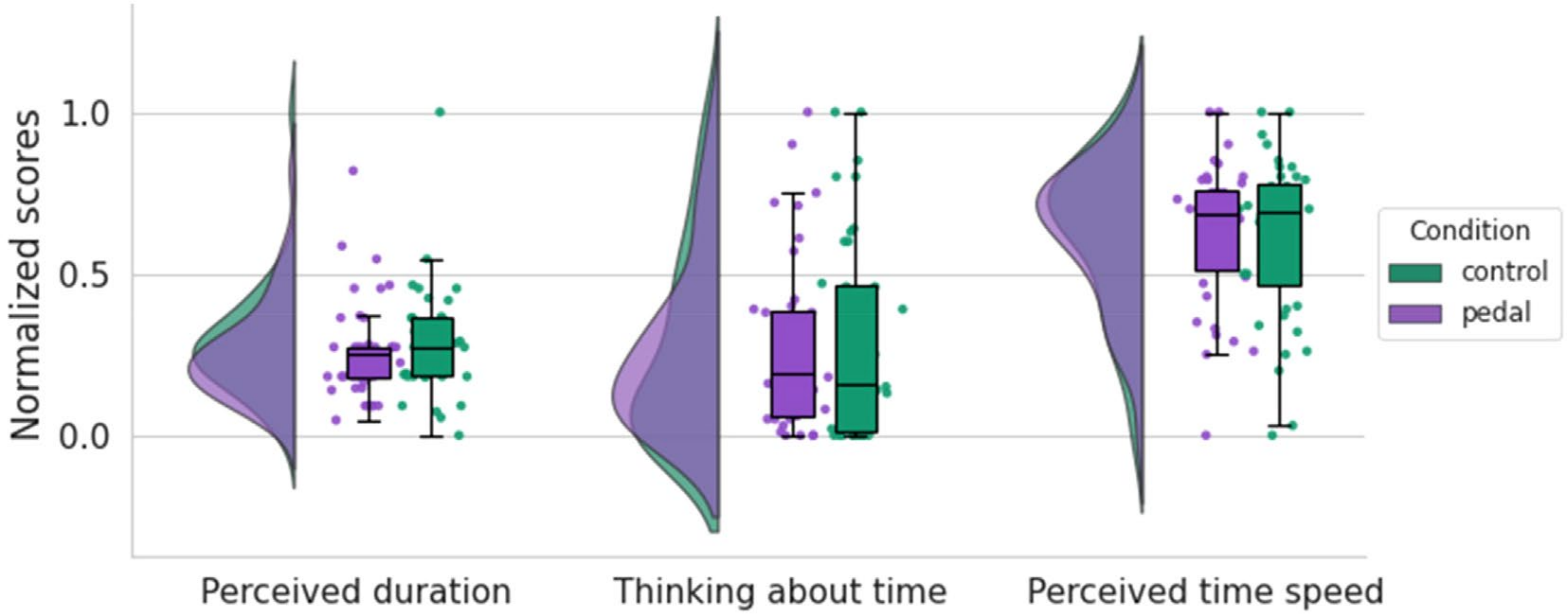
Descriptives of temporal measurements in control and pedal conditions. Normalized scores from Subjective Time, Self, and Space (STSS) questionnaire are displayed across two experimental conditions. Perceived duration in the control and pedal conditions indicates how long participants felt the gaming sessions lasted; Speed of time perception in each condition, which reflects how quickly or slowly participants felt time passed during the gaming sessions; The frequency of thinking about time in each condition, which reflects how often participants were consciously aware of the passage of time.

Further analysis revealed that the pedal did not influence participants' perception of time (assessed by STSS) across conditions, as evidenced by perceived duration (BF_01_ = 3.72, 95% CI = [−0.15, 0.45]), frequency of thinking about time (BF_01_ = 5.48, 95% CI = [−0.24, 0.35]), and speed of time passage (BF_01_ = 5.60, 95% CI = [−0.34, 0.25]). These findings provide support for Hypothesis 1b (Figure 4).

Moreover, we did not observe a considerable effect of the pedal on the body‐related scores: STSS bodily awareness (BF_01_ = 5.76, 95% CI = [−0.27, 0.33]), SAM emotional valence (BF_01_ = 5.78, 95% CI = [−0.32, 0.27]), SAM arousal (BF_01_ = 5.49, 95% CI = [−0.25, 0.35]), and ASC disembodiment (BF_01_ = 4.17, 95% CI = [−0.17, 0.42]). Other tests provided evidence for the null hypothesis, albeit with smaller evidence: ASC insightfulness (BF_01_ = 1.36, 95% CI = [−0.57, 0.41]) and ASC blissfulness scores (BF_01_ = 1.88, 95% CI = [−0.07, 0.54]).

We also explored the correlations between post‐task flow scores and other post‐task behavioral measures. In the control condition, post‐task flow scores were associated with a faster perceived passage of time (*r* = 0.59, BF_10_ > 30, 95% CI [0.32, 0.75]), reduced thinking about time (*r* = −0.61, BF_10_ > 30, 95% CI [−0.76, −0.35]), a smaller decrease in positive valence measured by SAM emotional valence change (*r* = −0.52, BF_10_ > 30, 95% CI [−0.70, −0.23]), increased ASC disembodiment (*r* = 0.40, BF_10_ = 4.40, 95% CI [0.09, 0.62]), and an increased ASC blissful state (*r* = 0.54, BF_10_ > 30, 95% CI [0.26, 0.71]).

In the pedal condition, post‐task flow scores were similarly correlated with the STSS time perception scores; notably, a faster time passage (*r* = 0.38, BF_10_ = 3.51, 95% CI [0.07, 0.61]), reduced thinking about time (*r* = −0.60, BF_10_ > 30, 95% CI [−0.75, −0.33]), a smaller decrease in positive valence (*r* = −0.44, BF_10_ = 9.96, 95% CI [−0.65, −0.14]), and an increased ASC blissful state (*r* = 0.42, BF_10_ = 7.31, 95% CI [0.12, 0.64]). Overall, the present results are consistent with the interpretation that the use of the pedal does not substantially interfere with the perceived degree of flow, body‐related experiences, or emotional states during flow, suggesting that it may function as a non‐disruptive tool. All robustness analyses and corresponding outputs are available in our OSF project.

### 3.2 Real‐Time Flow Ratings

**FIGURE 5:**
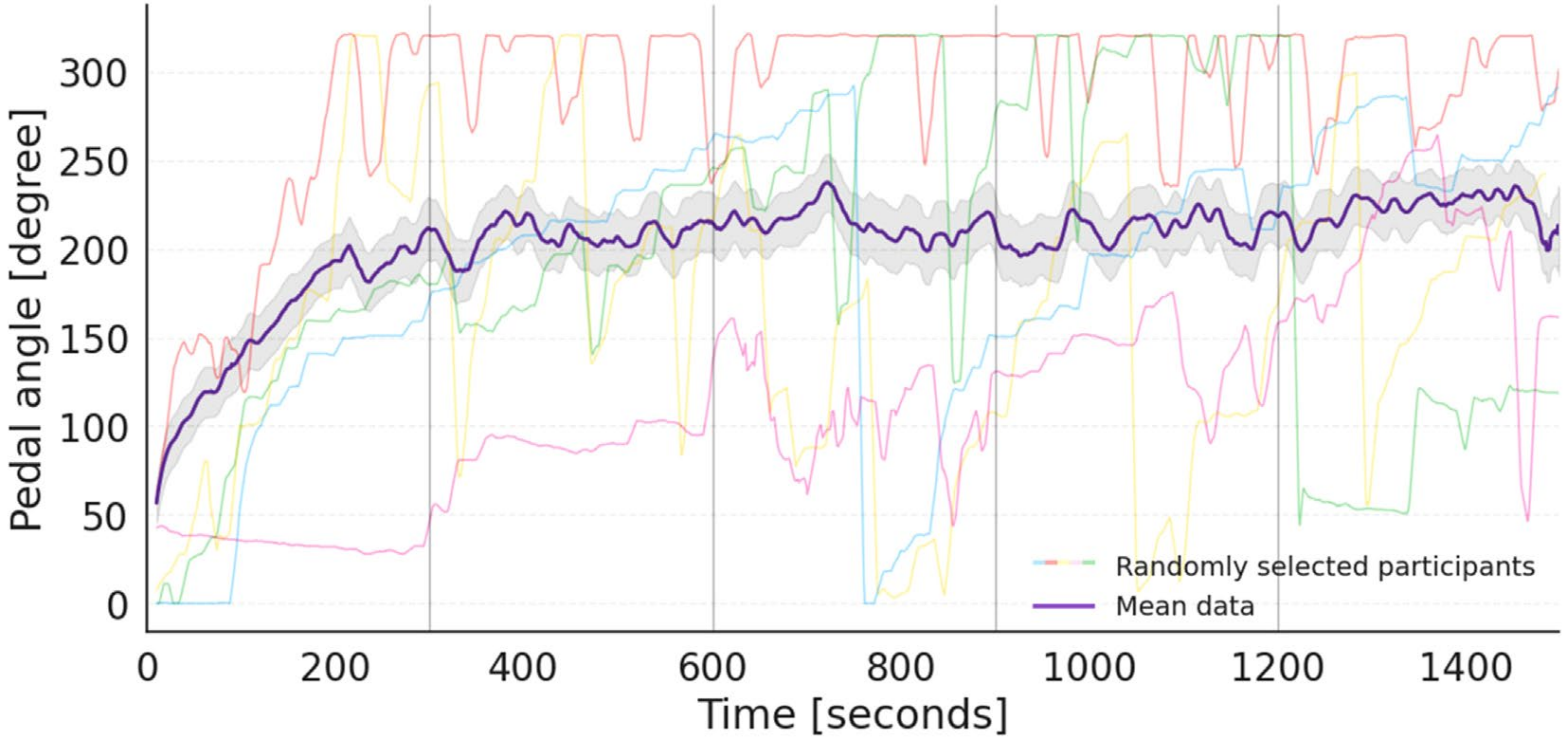
Descriptives of the pedal data. The purple line represents the mean real‐time flow ratings, with shaded areas indicating the standard error of the mean (SEM) averaged over 5‐min intervals across the 25‐min gaming session. Ratings were continuously recorded via a foot pedal (angle range: 0–324). The individual real‐time flow ratings of five randomly selected participants are also shown, each depicted in a different color.

Real‐time flow ratings, averaged across 5‐min epochs, revealed that participants pressed the pedal more frequently during the third, fourth, and fifth intervals, suggesting an increase in perceived flow as the session progressed (Figure 5). The real‐time ratings also demonstrated good internal consistency across the five epochs (*α* = 0.89, 95% CI = [0.83, 0.94]).

**FIGURE 6:**
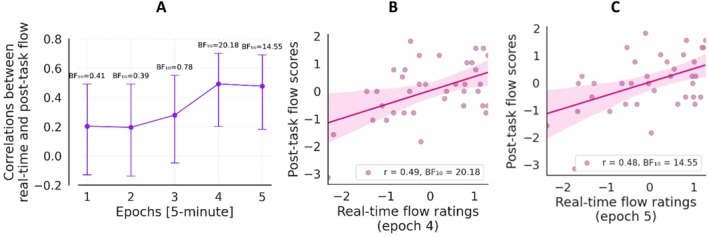
Correlations between real‐time and post‐task flow reports. (A) Results of the Bayesian correlation analyses between real‐time flow ratings and post‐task flow scores across five epochs, each representing a 5‐min interval within the 25‐min gaming session. (B) Correlation between real‐time flow ratings and post‐task flow scores in the 4th epoch. (C) Correlation between real‐time flow ratings and post‐task flow scores in the 5th epoch.

The subsequent research question explored whether the pedal reliably captures the flow experience (Hypothesis 2). We found strong evidence for a positive correlation between post‐task flow scores and the last 5 min of real‐time flow ratings (*r* = 0.48, BF_10_ = 14.55, 95% CI [0.17, 0.68]). Post‐task flow scores and the fourth epoch of real‐time flow ratings were correlated (*r* = 0.49, BF_10_ = 20.18, 95% CI [0.19, 0.69]). Correlations for each epoch of real‐time flow ratings can be seen in Figure 6, with particular emphasis on the final two epochs.

### 3.3 Physiological Measures

**FIGURE 7:**
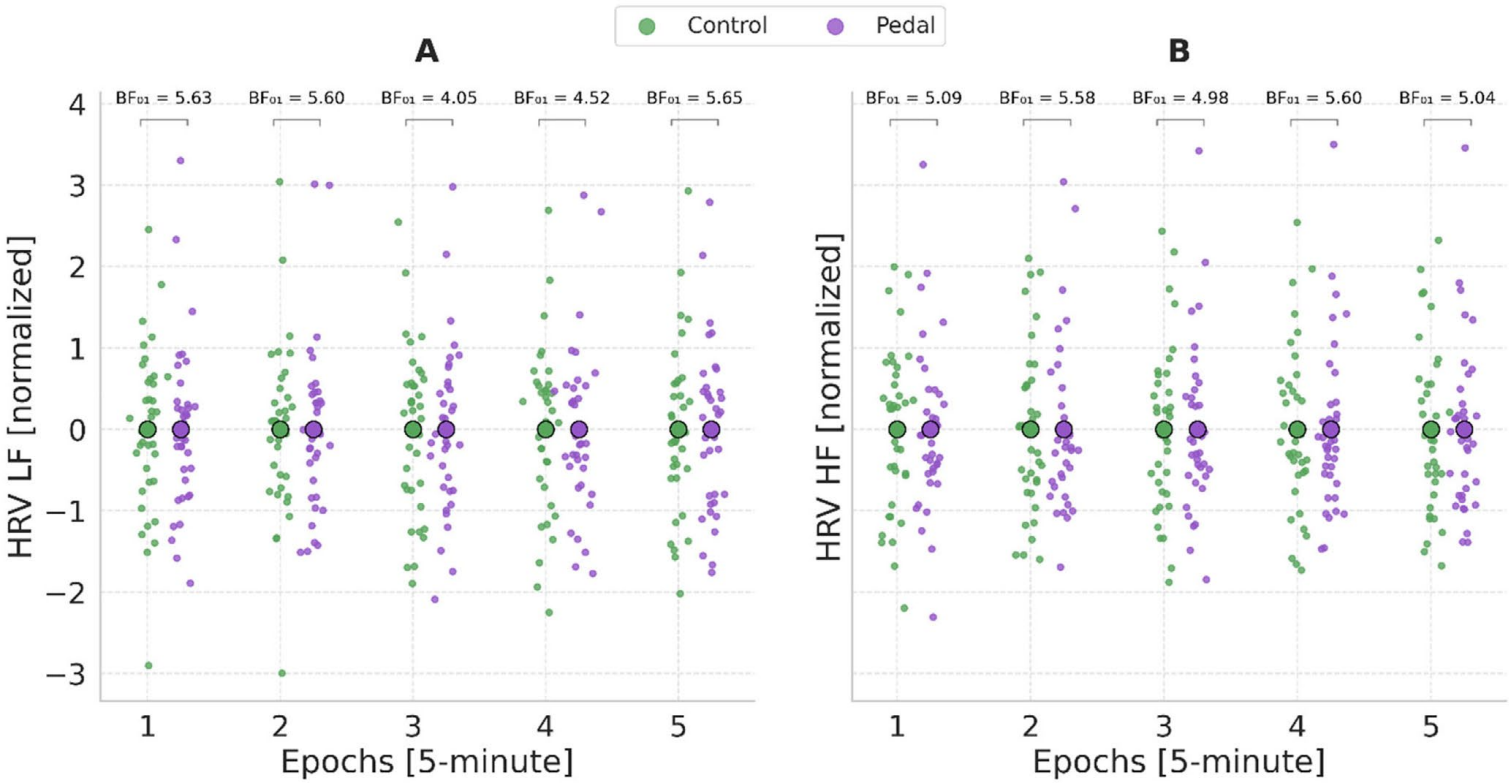
Distributions of LF‐HRV and HF‐HRV across 5‐min Epochs. Distributions of LF‐HRV (A) and HF‐HRV (B) values in pedal and control conditions across 5‐min epochs accompanied by Bayes Factors (BF_01_) from Bayesian paired samples *t*‐tests.

We compared physiological measurements over the 5‐min intervals across conditions to investigate whether they were affected by using the pedal. The comparison reveals that all epochs of LF‐HRV were similar (e.g., BF_01_ = 5.63, 95% CI = [−0.32, 0.29] during the first 5 min of both conditions). HF‐HRV levels were also similar across all epochs (e.g., BF_01_ = 5.09, 95% CI = [−0.24, 0.38] during the first 5 min of both conditions), supporting Hypotheses 3a and 3b (Figure 7). HF‐HRV and LF‐HRV trends across conditions are presented in Figure S2.

Under the narrower prior specification (Cauchy *r* = 0.1), Bayes factors remained in favor of the null hypothesis for HF‐HRV (BF_01_ ranging from 1.45 to 1.52 across epochs) and for LF‐HRV (BF_01_ ranging from 1.33 to 1.53 across epochs).

We calculated the changes in HRV metrics (difference between the last and first epochs) for each condition. The LF‐HRV changes in the control and pedal conditions were considerably similar (BF_01_ = 5.65, CI [−0.30, 0.31]). The HF‐HRV changes between conditions were also similar, although the evidence was weaker (BF_01_ = 2.66, CI [−0.51, 0.12]).

**FIGURE 8:**
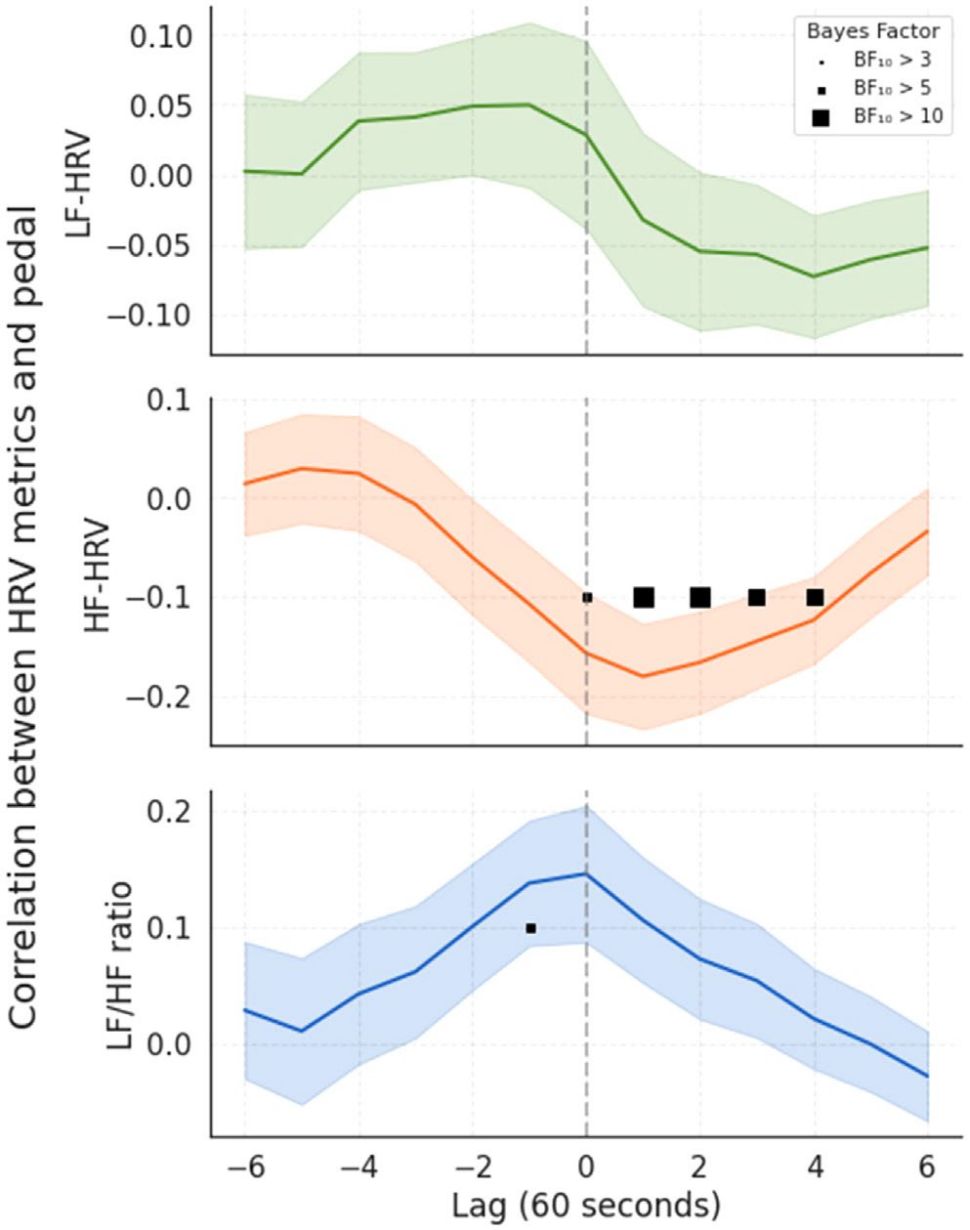
Cross‐correlation between pedal output and HRV metrics. Figure shows correlations between pedal data and HRV metrics (LF‐HRV, HF‐HRV and LF/HF ratio), with shaded error bands representing SEM. The Y‐axis represents correlations, while the X‐axis shows corresponding lags, with each unit representing 60 s. Results were visualized by marking time points where BF_10_ exceeded predefined thresholds (BF_10_ > 3, 5, or 10). Square markers of increasing size represent higher BF_10_ thresholds, providing a visual comparison of effect strength.

As a temporal analysis, we examined the relationship between HRV metrics (LF and HF power, and LF/HF ratio) and real‐time flow ratings (pedal data) using cross‐correlation with time lags ranging from −6 to 6 (each lag corresponds to 60 s). The results of this analysis are presented in Figure 8. In the correlation of HF‐HRV and pedal data, there was a negative relationship at and after lag 0. The peak effect was found at lag 1 (60s) with a correlation of −0.18 (BF_10_ = 19.02), indicating strong evidence for an association. All lags from 0 to 3 exceed BF_10_ > 3, indicating moderate to strong evidence for a negative relationship. The correlation between LF‐HRV and pedal data showed that all BF_10_ values were below the Bayes factor of 1, providing evidence against a correlation. The strongest, although without evidence, positive correlations were observed at lags −2 to 0, with mean correlations between 0.05 (BF_10_ = 0.28) and 0.03 (BF_10_ = 0.19). The correlation of LF/HF ratio and pedal data peaked at lag 0 (coefficient = 0.15, BF_10_ = 3.01) and lag −1 (coefficient = 0.14, BF_10_ = 2.59). These positive correlations with moderate evidence showed that a higher LF/HF ratio is associated with elevated flow.

Additionally, a linear mixed‐effects model was conducted to examine whether LF‐HRV, HF‐HRV, and their interaction predict pedal real‐time flow ratings. The model included a random intercept for participant ID to account for repeated measures across 24 windows per participant (*N* = 37; total observations = 888).

The analysis revealed a significant main effect of LF‐HRV [*F*(1, 884) = 7.23, *β* = 0.10, *p* < 0.05], indicating that a higher LF power was associated with increased pedal output. There was also a significant main effect of HF‐HRV [*F*(1, 884) = 27.07, *β* = −0.19, *p* < 0.001], indicating that higher HF power was associated with a decreased pedal angle. Their interaction was not statistically significant [*F*(1, 884) = 1.52, *β* = −0.04, *p* = 0.218]. Another linear mixed‐effects model conducted for the LF/HF ratio using the same number of observations showed that the LF/HF ratio was a significant predictor of pedal data [*F*(1, 884) = 17.37, *β* = 0.13, *p* = 0.001]. This showed that a higher LF/HF ratio was associated with elevated flow. Consistent with contemporary debates on its physiological interpretation (Thomas et al. 2019; Billman 2013), the LF/HF ratio is interpreted here as a broad indicator of autonomic balance rather than a specific marker of sympathetic dominance. Time‐based trends in the HF‐HRV and LF‐HRV, along with real‐time flow ratings, are presented in Figure S3; quantile‐based trends are presented in Figure S4.

**FIGURE 9:**
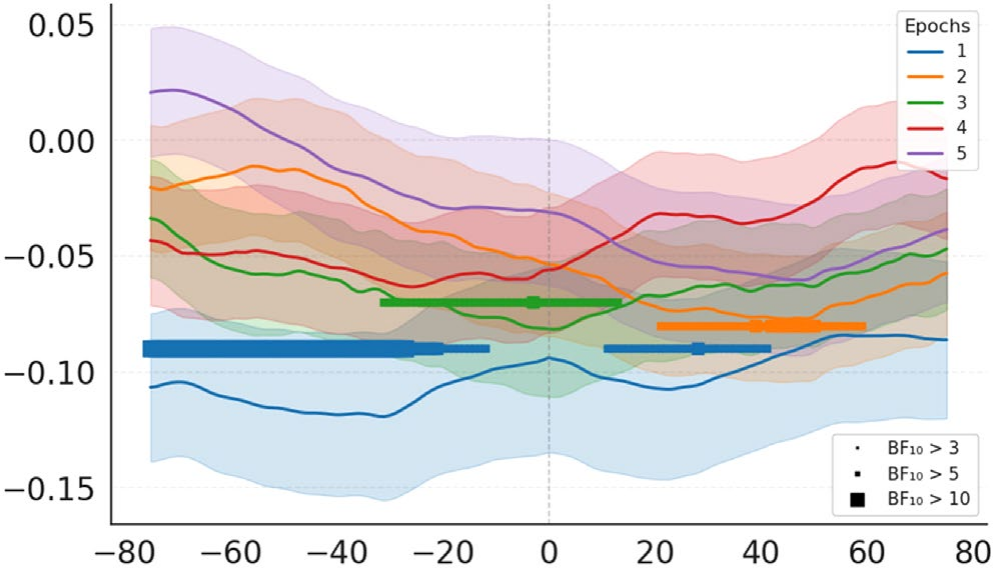
Cross‐correlation between pedal data and IBI. Figure shows correlations between pedal and IBI data across five epochs, with shaded error bands representing the SEM. The Y‐axis represents correlations, while the X‐axis shows corresponding lags with each unit representing 60 s. Results were visualized by marking time points where BF_10_ exceeded predefined thresholds (BF_10_ > 3, 5, or 10). Square markers of increasing size represented higher BF_10_ thresholds, providing a visual comparison of effect across epochs.

The mean IBI across five epochs ranged from 773.09 ms (SD = 155.04) in Epoch 1 to 752.90 ms in Epoch 5 (SD = 140.30). The trends in IBIs and real‐time flow ratings over the 25‐min game‐playing session can be seen in Figure S1. As an exploratory analysis, a cross‐correlation analysis was performed to investigate the temporal relationship between the IBI and real‐time flow ratings across five epochs (Figure 9).

In the first epoch, the maximum cross‐correlation coefficient was −0.12 at lag −32 (BF_10_ = 26), with 82.3% of all lags showing evidence for synchrony (BF_10_ > 3). In the second epoch, the peak correlation reached −0.08 at lag 45 (BF_10_ = 5.71), with 21.5% of lags exceeding the BF_10_ > 3 threshold. During the third epoch, the highest correlation was −0.08 at lag 1 (BF_10_ = 4.97), and 24.9% of lag values showed moderate evidence for synchrony (BF_10_ > 3). In epoch 4, the peak coefficient was −0.064 at lag −26 (BF_10_ = 1.75), with no lags exceeding BF_10_ > 3. However, 23.2% of lags showed weak evidence for an effect (BF_10_ > 1). In the fifth epoch, the peak correlation was −0.06 at lag 48 (BF_10_ = 0.85), and no lags exceeded the threshold.

## 4 Discussion

We introduced a novel method for continuously and effortlessly measuring perceived flow by using a foot pedal during gameplay in this study. The pedal recorded the angular position, with a full depression indicating a high level of flow and release corresponding to a lower flow level. This approach enabled real‐time and continuous reporting of the flow experience and the simultaneous co‐recording of subjective and physiological data. Participants played the game Thumper under two conditions while their ECG signals were recorded in a control condition (without the pedal) and an experimental condition (with the pedal). After each session, participants completed a series of post‐task questionnaires to assess their subjective experience.

### 4.1 The Pedal Did Not Disrupt the Flow Experience

The initial hypothesis postulates that using the pedal for real‐time flow reporting would not disrupt participants' flow experience (Hypothesis 1a), time perception (1b), bodily and spatial awareness (1c), valence and arousal levels (1d), or self‐consciousness (1e) during gameplay. Participants' average flow ratings in both conditions (4.90 in the pedal condition and 4.99 in the control condition on a 7‐point scale) were comparable to those reported by Khoshnoud et al. (2022), who found a mean score of 4.86 among non‐gamers while playing Thumper. Analyses comparing post‐task reports (i.e., FSS, STSS, SAM, and ASC) across conditions revealed no systematic differences in participants' ratings. Scores were comparable across conditions, suggesting that the use of the pedal did not disrupt the flow experience. Importantly, even under a conservative prior (*r* = 0.1), the evidence was still in favor of the null hypothesis. Together with our primary analyses (*r* = 0.7), these results suggest that the observed data are more compatible with no systematic difference between control and pedal conditions.

Similarly, related measures, such as sense of time, bodily and spatial awareness, arousal, and self‐consciousness, did not differ between conditions. These findings are consistent with the interpretation that integrating the pedal for real‐time subjective flow measurement may not substantially interfere with participants' overall subjective experience.

Post‐task behavioral measures have previously revealed that flow is marked by positive valence and heightened arousal (Kivikangas 2006). Individuals experience feelings of enjoyment during flow (Khoshnoud et al. 2020), combined with a certain level of stress due to intense mental effort (Peifer et al. 2014; Knierim et al. 2018). Our results also indicated that positive valence was sustained as flow increased, accompanied by an enhanced sense of bliss (as measured by ASC) in both conditions.

Another defining characteristic of flow is decreased time awareness, with individuals often reporting a faster perceived passage of time (Khoshnoud et al. 2022). In both conditions, higher levels of flow were associated with spending less time thinking about time and experiencing time as passing more quickly, corresponding with previously reported characteristics of the flow experience. These findings further demonstrate consistency across conditions.

### 4.2 The Pedal as a Reliable Measure of Flow

Consistent with Hypothesis 2, real‐time flow ratings during the second half of the task showed strong correlations with post‐task flow scores from the FSS validated by Rheinberg et al. (2003). This suggests that the pedal is a reliable tool for assessing flow in real time, as it captures subjective experiences that align closely with established questionnaire measures. By establishing the reliability of our tool, we wanted to bridge the gap between subjective experiences and physiological measurements, enabling a more comprehensive understanding of the flow experience.

Strong correlations between post‐task flow scores and real‐time flow ratings in the last ten, but not the first 10 min of the task coincide with the retrospective nature of post‐task reports. It is possible that participants primarily reported their experience in the latter part of the gaming session when completing the questionnaires. One potential interpretation, consistent with the peak‐end rule (Fredrickson and Kahneman 1993), is that retrospective evaluations may be influenced disproportionately by particularly immersive or final periods of the session. This especially emphasizes the importance of continuous subjective measures, which can capture the full trajectory of the experience, including earlier phases that may otherwise be overlooked.

Alternatively, the stronger correlations toward the end of the session may indicate an adaptation period related to pedal use. Participants may have needed more time to become proficient using the pedal effectively, resulting in more reliable flow ratings (i.e., better aligned with the scores from the established questionnaire) toward the end of the session. However, strong internal consistency between the epochs of real‐time flow ratings suggests that participants were consistent in their use of the pedal throughout the session. This indicates that any potential adaptation effect was limited.

Internal consistency of real‐time flow ratings across epochs was good both in the first sessions (i.e., when the pedal was used at the beginning of the experiment) and the second sessions of pedal usage (i.e., when the pedal was used after already completing a full gaming session).

### 4.3 The Pedal Did Not Affect Heart Rate Variability

Finally, consistent with Hypothesis 3, comparisons of LF‐HRV and HF‐HRV across five‐minute intervals revealed no systematic differences between conditions. Sensitivity analyses using a narrower prior (*r* = 0.1) yielded a comparable pattern of results, indicating that this conclusion was robust to prior specification, although the strength of evidence was reduced. Together, these findings suggest that reporting flow continuously via the pedal did not alter participants' autonomic nervous system activity. This is another validation point for our method, as both LF‐HRV and HF‐HRV have been linked to autonomic regulation during the flow experience (de Manzano et al. 2010; Keller et al. 2011; Harmat et al. 2015; Khoshnoud et al. 2022).

### 4.4 Temporal Relationship Between ANS Activity and Perceived Flow

The continuous flow ratings recorded throughout the gameplay enabled us to examine temporal trends in HRV metrics and real‐time flow ratings by conducting a cross‐correlation analysis. This analysis revealed distinct temporal dynamics between HRV components and the subjective flow experience. A robust negative association was observed between HF‐HRV and flow ratings, emerging at lag 0 and peaking at lag 1 (60 s later), suggesting that lower parasympathetic activity tends to co‐occur with, or follow shortly after, periods of higher reported flow.

In contrast, LF‐HRV showed no significant correlation, with all Bayes factors indicating evidence against an association. The LF/HF ratio showed a modest positive correlation with flow around lag 0 to −1, suggesting that relative shifts in autonomic balance are temporally aligned with periods of elevated flow. These temporal patterns should be interpreted as correlational rather than causal, reflecting the relative timing and co‐fluctuation of physiological and subjective measures.

de Manzano et al. (2010) and Gaggioli et al. (2013) suggested that flow is linked to an increase in the dominance of sympathetic activity marked by an increased LF/HF ratio. de Manzano et al. (2010) described the flow state as a heightened arousal state combined with a relaxed state, as demonstrated in deep breathing patterns. Our linear mixed‐effects model correspondingly revealed that the LF/HF ratio was a significant predictor of pedal data. However, the absence of a relationship with the LF‐HRV, combined with modest effects in the LF/HF ratio in the cross‐correlation analysis, implies that flow is primarily associated with a decrease in parasympathetic activity rather than broader sympathetic‐parasympathetic shifts.

The negative association between the HF‐HRV and pedal data in both the linear mixed‐effects model and the cross‐correlation analysis showed that reduced parasympathetic activity may be a more reliable physiological marker of perceived flow. However, several studies have suggested that LF power is one of the markers characterizing sympathetic activity during the flow state either with a decreased (Harmat et al. 2015; Tozman et al. 2015; Chanel et al. 2011) or moderate trend (Peifer et al. 2014; Bian et al. 2016).

To further explore the relationship between ANS activity and real‐time flow ratings, we conducted a cross‐correlation analysis between real‐time flow ratings and IBI data over five 5‐min epochs. Results revealed that the temporal association was strongest during the initial epochs of the gaming session and became less pronounced over time. During the first epoch, the strongest negative correlation between the IBI and pedal data was observed at lags between −31 and −35, corresponding to a time shift of approximately −7.75 to −8.75 s. This means that IBI values tended to precede pedal responses by approximately 8 s. Temporal alignment between physiological and subjective measures of flow occurred in 82.3% of lags in this epoch.

The correlations were modest in epochs 2 and 3, and the evidence was moderate. The direction of the alignment changed in epoch 2, and peak correlation occurred at a delay of approximately 11–12.25 s, meaning that changes in pedal responses tended to be followed by shifts in IBI. In epoch 3, a maximum IBI was found within ±0.5 s around the pedal response. This suggests a close temporal alignment between IBI and flow at this stage. Epochs 4 and 5 showed little to no evidence for a temporal relationship.

Taken together, these results point to a strong bottom‐up physiological influence on flow during the first 5 min of the task, possible top‐down modulation of physiological states in the second epoch, and a real‐time synchrony in the third epoch, where flow and ANS activity shift together. The weaker evidence during the last two epochs implied that the temporal association between the IBI and perceived flow diminished as the session progressed.

These combined findings show that the reported increase in sympathetic activity during flow (de Manzano et al. 2010; Bian et al. 2016) was evident in the earlier phases of flow experience. The shifts in the temporal direction of the relationship suggest that the ANS and flow dynamics are modulated across time, possibly reflecting changes between higher cognitive effort (de Sampaio Barros et al. 2018; Harris et al. 2017; Keller et al. 2011) and lower cognitive effort (Bian et al. 2016; Peifer et al. 2014). The negative correlations suggest that flow is accompanied by reduced parasympathetic influence. This aligns with previous studies conducted with various tasks, such as game playing (de Sampaio Barros et al. 2018), a computerized knowledge task (Keller et al. 2011), and daily tasks traced with the Experience Sampling Method (Gaggioli et al. 2013). Other studies demonstrated the opposite pattern in the IBI and flow relationship (Léger et al. 2014; Bian et al. 2016).

We demonstrated that our method is suitable for investigating the subjective experience of flow alongside physiological responses by testing hypotheses related to its reliability and validity. Previous research (Tozman et al. 2015; Castellar et al. 2016; de Manzano et al. 2010; Ulrich et al. 2016) usually relied on the common practice of pairing physiological data with self‐reports to examine flow. Physiological data recorded during task engagement are often interpreted with the help of post‐task reports obtained after task completion. Consequently, subjective assessments need to be correlated with physiological signals (Khoshnoud et al. 2020), as they provide the reference points for investigating how flow states vary across individuals and tasks.

However, the physiology of flow experience is difficult to correlate with current subjective measures (Harris et al. 2017), as the latter rely on retrospective assessments and provide non‐continuous data. These self‐reports cannot capture an accurate representation of the dynamic changes that occur throughout the task, as they only provide a broad summary of the experience. This limitation hinders a comprehensive understanding of the relationship between physiological responses and the subjective experience of flow while it unfolds during the experience. Csikszentmihalyi (1990) warns against over‐relying on these methods, fearing they might miss what makes flow such a unique experience.

### 4.5 Limitations and Future Directions

We did not record participants' game performance, which could have been informative for the pedal's influence on task execution. Performance measures would also have allowed us to examine the relationship between perceived flow and proficiency. Moreover, tracking performance over time could have helped identify signs of fatigue or fluctuations in engagement during the later stages of the session, providing a better understanding of behavioral outcomes associated with flow.

When employing proprietary games such as Thumper, rather than a custom‐designed experimental task, researchers has limited control over the task structure and data access as game developers may not always be willing or able to provide access to internal performance data (Allen et al. 2024). In particular, Thumper does not provide a detailed or continuous breakdown of gameplay performance (e.g., level‐by‐level scores, accuracy, or performance grades) that could be directly linked to real‐time subjective or physiological measures.

To assess the influence of the pedal on task execution, one would need to develop a dedicated annotation system capable of recording participants' gameplay dynamics throughout each session, including level progression, scores, and performance grades at high temporal resolution. Implementing such an annotation framework was beyond the scope of the present study. However, the development of such an annotation system should be planned for future studies to allow a more direct examination of how continuous flow ratings relate to task performance.

A further limitation is the homogeneity of gaming experience in the sample, which may limit the extent to which the results generalize to broader gaming populations. The sample consisted primarily of casual gamers, which may limit the extent to which the results generalize to broader gaming populations. Although Khoshnoud et al. (2022) showed that individuals with varied gaming backgrounds (i.e., gamers and non‐gamers) can experience similar levels of flow during Thumper, expert gamers may still engage with the pedal differently, potentially influencing real‐time flow ratings in our study. Future studies should therefore include a broader range of gaming profiles to examine whether the pedal operates similarly across levels of expertise and whether real‐time flow ratings are influenced by differences in skill level or familiarity with gameplay tasks.

An important limitation of the present study concerns the interpretation of the observed associations between real‐time flow ratings and post‐task flow reports. Although these correlations are consistent with the idea that the pedal captures moment‐to‐moment variations in perceived flow, they could also reflect a consistency process arising from individual response tendencies rather than genuine convergence on underlying flow experiences. Specifically, the association may be driven by the overall amount of pedal engagement rather than by the experiential content of the ratings themselves: participants who engaged more extensively with the pedal may also report higher post‐task flow, whereas those who engaged less may report lower flow, thereby shifting the relationship between real‐time and post‐task flow reports.

While the present data cannot fully rule out this possibility, two observations argue against a purely response‐tendency account. First, FSS scores did not differ between conditions with and without pedal use, suggesting that neither the presence nor the amount of using the pedal systematically influenced post‐task flow reports. Second, if pedal engagement reflected a general response tendency unrelated to flow, one would expect it to correlate broadly with other concurrent self‐report measures. However, pedal ratings were not associated with valence, arousal, bodily awareness, disembodiment, or time‐related experiences, providing some evidence for discriminant validity. We therefore interpret the association between pedal‐based ratings and FSS scores cautiously as initial evidence consistent with construct overlap, while emphasizing the need for future studies employing experimental manipulations of flow.

A possible experimental paradigm, as suggested by the reviewer, would address this concern by systematically increasing or decreasing flow (e.g., through manipulations of task difficulty or game design) and examining whether such changes are reflected in the relationship between real‐time flow ratings and post‐task reports. If such a manipulation alters both types of flow reports in the same direction, independently of the presence of the pedal, this would provide stronger evidence that the pedal tracks genuine changes in flow. Future studies could implement validated paradigms, such as the tile‐game task described by Melnikoff et al. (2022).

Another limitation of our study is the feasibility of the pedal. The gameplay task was performed while seated, which made it practical to use a foot pedal as a secondary tool for continuous flow reporting. However, this setup may not be suitable for other flow‐inducing activities, particularly those that are physically demanding or involve full‐body movement, such as sports, dance, or certain types of music performance.

Moreover, it remains unclear whether the strong internal consistency observed in our VR gaming context would hold in tasks that rely on different sensory modalities or cognitive demands. Future research should therefore examine the applicability of the pedal‐based method across a wider range of task types or develop alternative input devices that can provide comparable real‐time flow measurements in settings where a foot‐operated mechanism is impractical. Such research is essential for enabling continuous flow reporting across diverse domains.

Given the novelty of our method, further validation studies are necessary. This method permits the collection of continuous data that can be integrated with physiological measures to investigate the dynamics of the flow. For example, patterns in physiological signals alongside real‐time flow ratings may help identify distinct phases of the experience. We used HRV metrics to assess the physiological effects of our method and to explore the dynamic relationship between flow experience and autonomic regulation. However, while HRV provides information relating to autonomic regulation, the interpretation of specific indices remains a subject of debate.

HRV is widely accepted as a valid indicator of parasympathetic function, but its ability to capture sympathetic activity is contentious (Quigley et al. 2024; Thomas et al. 2019; Billman 2013). The primary issue is that the relationship between the autonomic branches is non‐linear, and parasympathetic activity often influences the low‐frequency spectrum, thereby confounding the interpretation of sympathetic dominance (Thomas et al. 2019). This indicates that relying on the LF/HF ratio as an indicator of the predominance of sympathetic activity is problematic. Therefore, findings involving the LF/HF ratio should be interpreted cautiously.

Consequently, cardiac autonomic markers may lead to an incomplete picture of the accompanying physiological processes. Future research should therefore incorporate additional measures, such as skin conductance or respiration, to capture autonomic engagement during flow. Furthermore, neurophysiological measures, such as EEG signals, should also be incorporated in future studies to explore the cognitive and neural processes associated with flow parallel to real‐time subjective data.

Finally, the present study relied on a single‐session design. Because participants used the pedal only once, we could not examine how they adapt to using it throughout the task. Repeated‐session or longitudinal designs would allow future research to assess how participants' use of the pedal changes over time, and whether the consistency of real‐time flow reporting improves with repeated use. Incorporating multiple or longer sessions with pedal usage would therefore provide stronger evidence for the usability of the pedal.

In conclusion, we introduced and validated a novel method for continuously measuring perceived flow in real time. Our findings demonstrate that the pedal may provide a practical and reliable tool for tracking subjective flow online without interfering with the experience itself.

## Author Contributions

**Sura Genc:** conceptualization, methodology, software, data curation, investigation, validation, formal analysis, visualization, project administration, writing – original draft, writing – review and editing. **Elif Surer:** supervision, writing – review and editing. **Marc Wittmann:** validation, supervision, resources, review and editing. **Tzvetan Popov:** conceptualization, validation, supervision, project administration, resources, writing – review and editing. **Bigna Lenggenhager:** conceptualization, validation, supervision, funding acquisition, project administration, resources, writing – review and editing.

## Conflicts of Interest

The authors declare no conflicts of interest.

## Supporting information


**Figure S1:** Trends in inter‐beat intervals and real‐time flow ratings. Standardized inter‐beat intervals (IBIs; in milliseconds) and real‐time flow ratings across the 25‐min gaming session in the pedal condition. Both time series were smoothed using 25 Hamming windows. The standard deviations displayed above the figure reflect values adjusted across epochs to account for between‐subject differences.
**Figure S2:** Trends in heart rate variability across conditions. Log transformed power of high‐frequency (HF) and low‐frequency (LF) heart rate variability (HRV) metrics across 25 sliding windows in the pedal and control conditions.
**Figure S3:** Trends in heart rate variability and real‐time flow ratings. Standardized high‐frequency (HF) and low‐frequency (LF) heart rate variability (HRV) metrics, along with real‐time flow ratings (pedal data), are shown for the pedal condition. HRV values were computed across 25 sliding windows, represented by dots along the LF (blue) and HF (orange) lines. Pedal data (purple) was visualized using 25 Hamming windows to show detailed fluctuations in the signal.
**Figure S4:** Heart rate variability across ten quantiles of pedal data. The distribution of standardized values of high‐frequency (HF), low‐frequency (LF) heart rate variability (HRV) metrics and their ratio (LF/HF HRV) across 10 quantiles of standardized pedal angle values. The quantiles were extracted from 5‐min intervals of the dataset.

## Data Availability

This manuscript is publicly available as a preprint on the Open Science Framework (OSF): https://osf.io/preprints/osf/ex5mr_v1. The data and analysis scripts are publicly accessible via the OSF at: https://osf.io/p7ydv/.
