# Supplementary material for: Tracking Flow in Real Time: Continuous Measurement of Game‐Induced Flow in Virtual Reality
Source: Psychophysiology. 2026 Apr 7;63(4):e70283. doi: 10.1111/psyp.70283 (PMC13057059; doi:10.1111/psyp.70283)
Supplement: Supplementary file 1 — Figure S1: Trends in inter‐beat intervals and real‐time flow ratings. Standardized inter‐beat intervals (IBIs; in milliseconds) and real‐time flow ratings across the 25‐min gaming session in the pedal condition. Both time series were smoothed using 25 Hamming windows. The standard deviations displayed above the figure reflect values adjusted across epochs to account for between‐subject differences. Figure S2: Trends in heart rate variability across conditions. Log transformed power of high‐frequency (HF) and low‐frequency (LF) heart rate variability (HRV) metrics across 25 sliding windows in the pedal and control conditions. Figure S3: Trends in heart rate variability and real‐time flow ratings. Standardized high‐frequency (HF) and low‐frequency (LF) heart rate variability (HRV) metrics, along with real‐time flow ratings (pedal data), are shown for the pedal condition. HRV values were computed across 25 sliding windows, represented by dots along the LF (blue) and HF (orange) lines. Pedal data (purple) was visualized using 25 Hamming windows to show detailed fluctuations in the signal. Figure S4: Heart rate variability across ten quantiles of pedal data. The distribution of standardized values of high‐frequency (HF), low‐frequency (LF) heart rate variability (HRV) metrics and their ratio (LF/HF HRV) across 10 quantiles of standardized pedal angle values. The quantiles were extracted from 5‐min intervals of the dataset. [file PSYP-63-e70283-s001.docx]

# Supplementary Material

***Real-Time Tracking of Flow: Continuous Measurement of Game-Induced Flow in Virtual Reality***

*Sura Genc, Elif Surer, Marc Wittmann, Tzvetan Popov, Bigna Lenggenhager*

**Supplementary Figure 1**

*Trends in Inter-Beat Intervals and Real-Time Flow Ratings*

**
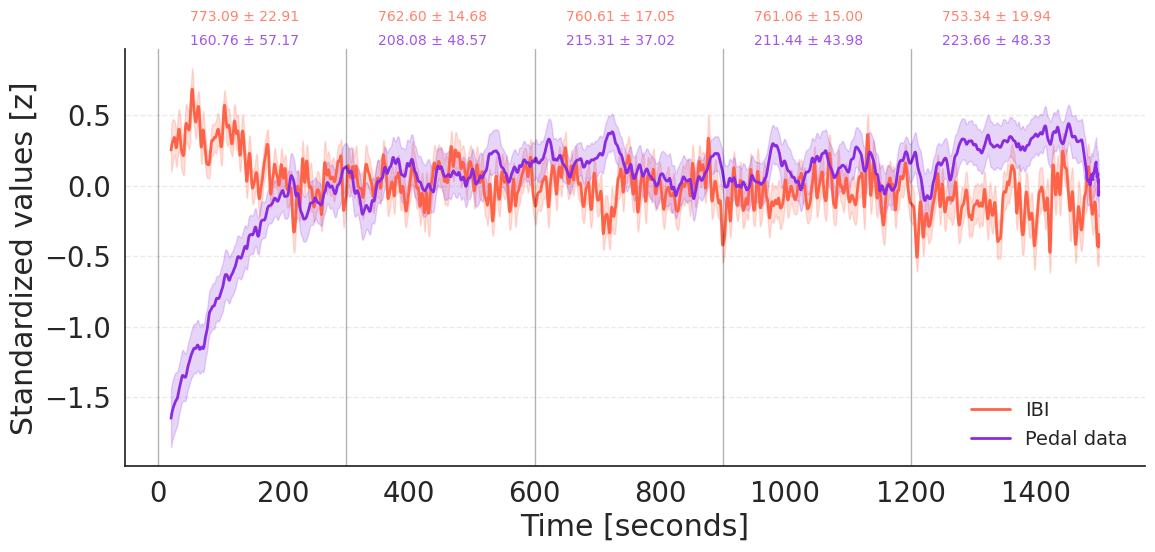
**

*Note.* Standardized inter-beat intervals (IBIs; in milliseconds) and real-time flow ratings across the 25-minute gaming session in the pedal condition. Both time series were smoothed using 25 Hamming windows. The standard deviations displayed above the figure reflect values adjusted across epochs to account for between-subject differences.

**Supplementary Figure 2**

*Trends in Heart Rate Variability Across Conditions*

*
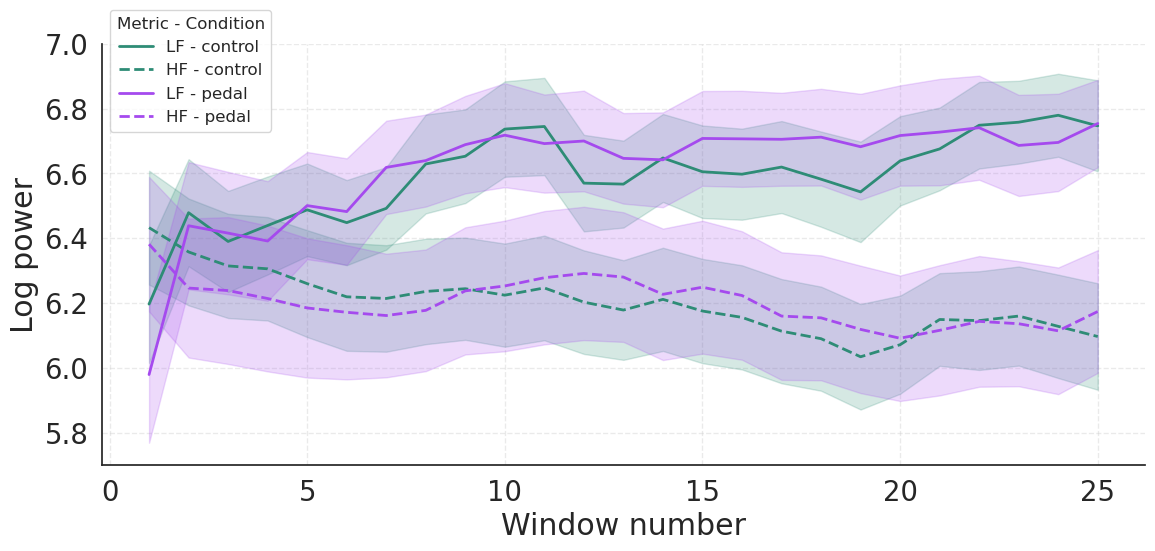
*

*Note.* Log transformed power of high-frequency (HF) and low-frequency (LF) heart rate variability (HRV) metrics across 25 sliding windows in the pedal and control conditions.

**Supplementary Figure 3**

*Trends in Heart Rate Variability and Real-Time Flow Ratings*

**
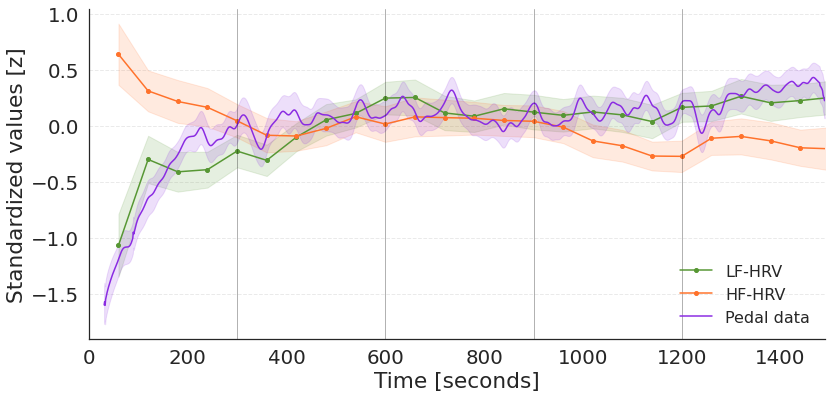
**

*Note.* Standardized high-frequency (HF) and low-frequency (LF) heart rate variability (HRV) metrics, along with real-time flow ratings (pedal data), are shown for the pedal condition. HRV values were computed across 25 sliding windows, represented by dots along the LF (blue) and HF (orange) lines. Pedal data (purple) was visualized using 25 Hamming windows to show detailed fluctuations in the signal.

**Supplementary Figure 4**

*Heart Rate Variability Across Ten Quantiles of Pedal Data*

*
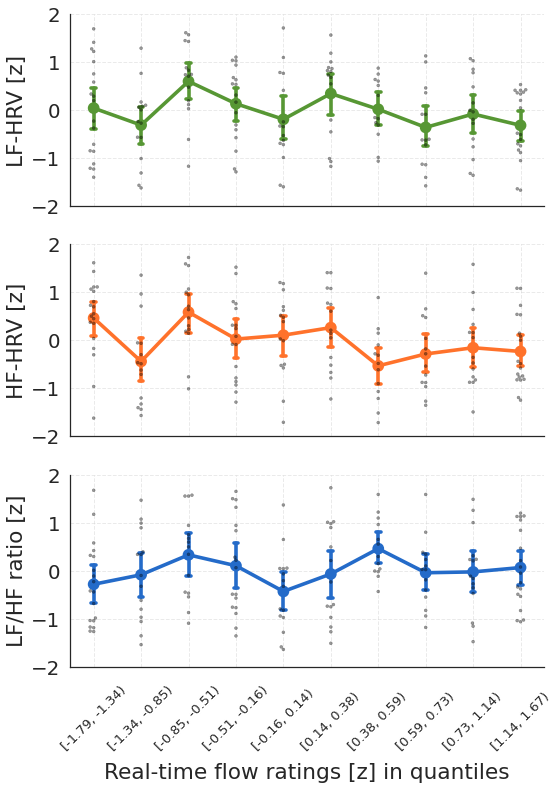
*

*Note.* The distribution of standardized values of high-frequency (HF), low-frequency (LF) heart rate variability (HRV) metrics and their ratio (LF/HF HRV) across 10 quantiles of standardized pedal angle values. The quantiles were extracted from 5-minutes intervals of the dataset.
